# Supplementary material for: Assessing the Efficacy of a Brief Universal Family Skills Programme on Violence and Substance-Use Indicators in Youth in Trentino and Parma, Italy: Study Protocol for a Multi-Centre, Non-Blinded, Cluster-Randomised Controlled Trial (cRCT) of Family UNited
Source: Int J Environ Res Public Health. 2023 Aug 8;20(16):6548. doi: 10.3390/ijerph20166548 (PMC10454720; doi:10.3390/ijerph20166548)
Supplement: Supplementary file 1 [file ijerph-20-06548-s001.zip › ijerph-2436700-supplementary.pdf]

Supplementary Materials

Informed consent/assent material in English

Assessing the effectiveness and acceptability of Family UNited – Italy

CONSENT FORM - Questionnaires

If you are willing to participate please complete and sign the consent form below.

Please initial box

1.	I confirm that I have read the information sheet on the above project and have had the opportunity to consider the information and ask questions and had these answered satisfactorily.	
2.	I understand that my participation in the study is voluntary and that I am free to withdraw at any time without giving a reason and without it affecting any other treatment or service.	
3.	I understand that data collected may be used in future studies and will be stored confidentially for a minimum of 5 years and will then be destroyed.	
4.	I understand that my name or any identifying material will not be used and we will not be personally identified in any reports or publications of the research.	
5.	I agree to take part in the above project.	

Name of participant ____________ Date ____________ Signature ____________

Name of person taking consent ____________ Date ____________ Signature ____________

Assessing the effectiveness and acceptability of Family UNited – Italy

ASSENT FORM (CHILD)

Child (or if unable parent on their behalf) to initial in the boxes and sign below

Please tick the box
If you agree

1.	I have read the information about this project or someone has read it to me.	
2.	Somebody has explained the project to me.	
3.	I understand what this project is about.	
4.	I understand what I will be asked to do in this project.	
5.	I have asked all the questions I want to.	
6.	I have had all my questions answered and understand.	
7.	I understand that it's OK to stop taking part at any time.	
8.	I agree to take part in the above project.	

Name of participant	Date	Signature
Name of person taking assent	Date	Signature

Informed consent/assent material in Italian

INFORMATIVA PER IL TRATTAMENTO DI DATI IN UN PROGETTO DI RICERCA

Lei è invitata / invitato a prendere parte ad uno studio che si propone di validare un progetto di miglioramento della coesione, della comunicazione e delle relazioni familiari, nonché delle abilità di regolazione emotiva e abilità assertive dei minori, condotto nel Dipartimento di Medicina e Chirurgia, in collaborazione con la Comunità Betania di Parma, l'Università degli Studi di Trento e sotto la supervisione della Sezione UNODC delle Nazioni Unite. Le caratteristiche dello studio e le sue modalità di svolgimento sono descritte nel dettaglio nel documento "*Consenso informato alla partecipazione alla ricerca- adulti/minori*".

SOGGETTI DEL TRATTAMENTO

L'Università degli Studi di Parma con sede in Parma Via Università 12 Cap. 43121 Parma (Tel. +390521.902111 – pec: protocollo@pec.unipr in qualità di Titolare del trattamento, tratterà i Suoi dati personali soltanto nella misura in cui siano indispensabili in relazione all'obiettivo dello studio, nel rispetto di quanto previsto dalla normativa vigente in materia di protezione dei dati personali: Regolamento UE 2016/679 (GDPR), D.lgs n. 196/2003 "Codice in materia di protezione dei dati personali" come modificato dal D.lgs n. 101/2018 e conformemente alle disposizioni di cui alle autorizzazioni generali dell'Autorità Garante per la protezione dei dati personali.
Il Responsabile della ricerca è Annalisa Pelosi, domiciliato per la sua carica presso il Dipartimento di Medicina e Chirurgia, Unità di Neuroscienze, v. Volturno 39, Parma.
Il Responsabile per la Protezione dei dati (RPD-DPO) cui potrà rivolgersi per questioni relative al trattamento dei Suoi dati personali, raccolti nell'ambito dello Studio oggetto della presente informativa e all'esercizio dei diritti ivi connessi è contattabile ai seguenti recapiti: dpo@pec.unipr.it
Ai sensi della normativa sopra citata il trattamento dei Suoi dati personali da parte dei soggetti autorizzati al trattamento coinvolti nello Studio, sarà improntato al rispetto dei principi di cui all'art. 5 del GDPR e, in particolare a quelli di liceità, correttezza, trasparenza, pertinenza, non eccedenza e in modo da garantire un'adeguata sicurezza dei dati personali.

FINALITÀ DEL TRATTAMENTO E BASE GIURIDICA

I dati da Lei forniti saranno trattati per finalità di ricerca applicata nell'ambito della psicologia della famiglia e della prevenzione universale. Il trattamento dei Suoi dati personali viene effettuato dal Titolare nell'ambito di esecuzione dei propri compiti di interesse pubblico ai sensi dell'art. 6, paragr. 1 lett. e) del GDPR. Il trattamento delle categorie particolari di dati personali (dati sensibili) viene effettuato per fini di ricerca scientifica ai sensi dell'art. 9, paragr. 2 lett. a) del GDPR sulla base di un consenso esplicito da Lei prestato al trattamento di tali dati personali che ha, in ogni caso, il diritto di revocare in qualsiasi momento senza incidere sui trattamenti già effettuati.

TIPOLOGIA DI DATI E MODALITÀ DEL TRATTAMENTO

I dati saranno trattati mediante strumenti cartacei ed elettronici. I ricercatori che seguiranno lo studio identificheranno l'interessato con un codice. I dati raccolti nel corso dello studio saranno registrati, elaborati e conservati per il tempo necessario alle finalità scientifiche del presente studio (orientativamente, due anni a partire dalla conclusione della raccolta dei dati). Soltanto il ricercatore ed i soggetti autorizzati potranno collegare questo codice al Suo nominativo. I dati saranno diffusi solo in forma aggregata e rigorosamente anonima, ad esempio attraverso pubblicazioni scientifiche, statistiche e convegni scientifici.

NATURA DEL CONFERIMENTO DEI DATI

Il conferimento pur essendo facoltativo è necessario per le suddette finalità di ricerca, è indispensabile per lo svolgimento dello Studio e non discende da un obbligo normativo. Il mancato conferimento determina l'impossibilità di partecipare allo Studio.

DIRITTI DELL'INTERESSATO

Nella sua qualità di Interessato, il soggetto partecipante allo studio in parola gode dei diritti di cui alla sezione 2, 3 e 4 del capo III del Regolamento (UE) 2016/679 (es. chiedere al titolare del trattamento: l'accesso ai dati personali e la rettifica o la cancellazione degli stessi; la limitazione del trattamento che lo riguardano; di opporsi al loro trattamento).
L'Interessato ha altresì il diritto di:

- revocare il consenso in qualsiasi momento senza tuttavia pregiudicare la liceità del trattamento basata sul consenso prestato prima della revoca. In tal caso, non saranno raccolti ulteriori dati che lo riguardano, ferma restando l'utilizzazione di quelli eventualmente già raccolti per determinare, senza alterarli, i risultati della ricerca o di quelli che, in origine o a seguito di trattamento, non siano riconducibili a una persona identificata o identificabile.
- proporre reclamo a un'autorità di controllo.

In merito all'esercizio di tali diritti, l'Interessato può rivolgersi al Responsabile locale del trattamento i cui recapiti sono indicati nel primo paragrafo della presente informativa e al DPO.

CONSENSO

Acconsento al trattamento dei dati per le finalità dello Studio descritte nella presente informativa, nei limiti e con le modalità ivi indicate.
Al Titolare è fatto divieto di utilizzare le immagini raccolte secondo modalità che ne pregiudichino la dignità personale, professionale e il decoro. L'acquisizione delle immagini

Nome e Cognome dell'interessato/a (in stampatello) ___________________________
Nato/a _______________________________ il ___________________
Firma dell'interessato _______________________________

Data _____________

CONSENSO MINORI

Il/la sottoscritto/a ________________________________ nato/a a _______________________ il ________________, residente in via _____________________ città______________________ prov. _____ e
Il/la sottoscritto/a _______________________________ nato/a a _______________________ il ________________, residente in via _____________________ città______________________ prov. _____

GENITORI/TUTORI LEGALI del minore ____ nato/a______________________ il ________________, residente a _____________________

Acconsento al trattamento dei dati per le finalità dello Studio descritte nella presente informativa, nei limiti e con le modalità ivi indicate.

Firma del/dei genitore/i o tutore/i _______________________________

Participant information sheet in English

Assessing the effectiveness and acceptability of Family UNited – Italy

Participant Information Sheet-Caregiver

You are being invited to take part in a research study. Please read this information sheet and discuss it with others if you wish. Take time to decide whether or not you wish to take part. Thank you for reading this.

Who will conduct the research?

The study will be run by a team of researchers from The United Nations Office on Drugs and Crime (UNODC) in cooperation with the University of Parma and the University of Trento. The group conducts research to improve the wellbeing of children and families as part of the ongoing programming to support families in Italy.

Title of the Research

Assessing the effectiveness and acceptability of Family UNited in Trento and Parma, Italy.

What is the aim of the research?

Parents and caregivers are very important in protecting children against emotional and behavioural challenges. Family factors play a large part in children and youths' ability to overcome challenging situations and achieve positive future outcomes. But often parents that have experienced challenges or difficult family circumstances, say they want help and advice in how to care for their children. We would like to help families by offering them parenting knowledge and skills that helps families function better and reduces stress. The aim of this research is to find out if the Family UNited Programme helps reduce problem behaviour in children and improves family functioning in families in Italy.

Why have I been chosen?

You have been chosen because you are a parent or caregiver, currently residing in Trento or Parma, Italy.

What would I be asked to do if I took part?

Participation in the Family UNited programme includes the attendance of 4 sessions for caregivers and 4 sessions for children. In addition, there will be 4 sessions in which caregivers and children will attend together, in a so-called family session. Each session

is one hour long and takes place in groups of around 12 families. You will learn about issues such as understanding stress, using love and affection in the family and also setting limits and dealing with disobedience.

You will also be asked to attend group assessments (1) prior to the first session of the programme, (2) two weeks after the programme, and (3) six weeks after the programme. During the assessments the researcher will ask you to complete some questions about your child's behaviour, how you have been feeling and how you have found parenting your child. You will also be asked some general questions about your family, such as your age and how many children you have.

What happens to the data collected?

The data collected will be stored confidentially for a period of 5 years.

Will my data be confidential?

Yes, all data collected will remain confidential at all times. Your data will be labelled with an identification number and not your name and will be kept confidentially and securely using this number. The only people who will have access to your individual identity and your data are the research team. The only exception to your data not remaining confidential would be if you disclose information that gives the researcher(s) reason to believe that you or your child's safety is at risk. If this happened, we may be duty bound to tell someone from outside of the research team this information but you would also be informed should this be the case.

What happens if I do not want to take part or if I change my mind?

It is up to you to decide whether or not to take part. If you do decide to take part, you will be given this information sheet to keep and be asked to sign a consent form. If you decide to take part you are still free to withdraw at any time without giving a reason and without detriment to yourself.

Will I be paid for participating in the research?

No. You will not be paid for taking part on this research, but will be offered to cover the cost of your transport to attend the 2 meetings.

What is the duration of the research?

You will be enrolled in the study for 3 months.

Where will the research be conducted?

The research will be conducted in the same place that the Family UNited Programme was running.

Will the outcomes of the research be published?

The research will be used to develop future studies and in delivering the programme to other families. It will be presented at national and international conferences and may

be published in academic journals. You will not be personally identified in any reports about the research.

Contact for further information

If you have a concern about any aspect of the study, you can contact Dr. Riccardo Lodi, riccarlodi@gmail.com; (for the group of families living in the area of Parma) or Dr. Valentina Molin, valetinamolin@yahoo.it; (for the group of families living in Trento).

What if I have further questions about my child?

If you need further advice regarding your child behaviour, you should contact your local doctor or community health centre.

Participant information sheet in Italian

CONSENSO INFORMATO ALLA PARTECIPAZIONE ALLA RICERCA – ADULTI

1. **Titolo, Responsabile progetto**

Gentile Signora/Signore,
Lei è invitata/o a prendere parte a una ricerca condotta nel quadro del progetto "Validazione italiana con studio multicentrico RCT del progetto Family UNited per il rafforzamento delle competenze familiari", di cui è responsabile la dott.ssa Annalisa Pelosi, Dipartimento di Medicina e Chirurgia, Unità di Neuroscienze (Ateneo di Parma).
Prima di decidere se partecipare è importante che abbia tutte le informazioni necessarie per aderire in modo consapevole e responsabile. Le chiediamo di leggere questo documento e di porre a chi le ha proposto questo studio tutte le domande che ritiene opportune.

2. **Breve descrizione e Obiettivi**

Il progetto di ricerca "Validazione italiana con studio multicentrico RCT del progetto Family UNited per il rafforzamento delle competenze familiari" persegue l'obiettivo di validare un progetto di miglioramento della coesione, della comunicazione e delle relazioni familiari, nonché delle abilità di regolazione emotiva e abilità assertive dei figli. Per raggiungere questo obiettivo, i ricercatori impegnati nel progetto si propongono di coinvolgere piccoli gruppi di genitori/caregiver e minori, di età compresa tra 8 e 15 anni, in quattro incontri a cadenza settimanale: nella prima ora di ogni incontro due facilitatori, opportunamente formativi, lavoreranno con i genitori/caregivers per imparare a conoscere il comportamento dei bambini e capire come l'attenzione del caregiver cambi il comportamento dei bambini, apprendere tecniche per facilitare la manifestazione dei comportamenti desiderati nei bambini e per comunicare con i loro figli promuovendone l'ascolto e la percezione di sentirsi sicuri e amati. In una diversa stanza, altri due facilitatori interagiranno con i bambini/ ragazzi, che apprenderanno modi positivi per affrontare lo stress e l'importanza di parlare dei propri sentimenti con adulti fidati, riflettendo sulle responsabilità di chi si prende cura di loro e delle qualità positive che vogliono sviluppare; inoltre, i bambini apprenderanno a gestire le situazioni di pressione da parte dei coetanei. Nella seconda ora, tutti i facilitatori, i genitori/ caregiver e i minori si riuniranno nella stessa stanza per la sessione familiare, in cui potranno mettere in pratica una comunicazione positiva e rispettosa, facendo giochi ed attività che incoraggiano una relazione positiva e creano fiducia.

3. **Che cosa comporta la partecipazione allo studio?**

La partecipazione al progetto di ricerca "Validazione italiana con studio multicentrico RCT del progetto Family UNited per il rafforzamento delle competenze familiari" comporta, oltre alla partecipazione alle quattro sedute del programma descritte nel punto precedente, la compilazione di test standardizzati: per i genitori, è prevista la rilevazione di alcune caratteristiche anagrafiche, lo screening delle modalità comportamentali dei bambini/ragazzi, la rilevazione della fiducia nelle proprie competenze genitoriali e della gestione dello stress. I minori compileranno scale di rilevazione della resilienza e dell'atteggiamento verso la violenza interpersonale tra pari. Tutti gli strumenti saranno compilati online, prima di iniziare il progetto, dopo due e sei settimane dal termine del progetto. A intervento concluso, su base volontaria, alcuni partecipanti potranno partecipare anche a un'intervista semi-strutturata, condotta da personale esterno alla conduzione del progetto, relativa alla verifica del miglioramento del benessere familiare e all'applicabilità quotidiana delle tecniche, delle metodologie e degli

strumenti appresi; in questa occasione potranno essere espresse eventuali criticità e suggerimenti.

4. **Benefici, disagi e/o rischi potenziali della partecipazione**

La partecipazione allo studio è volontaria e a titolo gratuito. Per i genitori / caregiver e i minori partecipanti la collaborazione non comporta nessun tipo di rischio; è tuttavia possibile che durante le prime sessioni del programma alcuni genitori/caregiver possano sperimentare transitori sentimenti di ansia o inadeguatezza riflettendo sul proprio ruolo e sulle modalità in cui viene esercitato. Queste reazioni sono previste e i formatori sono addestrati ad accoglierle e a gestirle come parte integrante del protocollo stesso: la diade genitore/figlio può sperimentare concretamente, in ciascuna sessione del progetto, le modalità di essere un attore positivo nella co-costruzione della propria relazione familiare, facilitando il superamento di insicurezze e consolidando un'immagine positiva di sé nel proprio ruolo familiare o disagio. I ricercatori si attendono informazioni sul miglioramento delle competenze familiari e della qualità della vita relazionale ed emotiva della famiglia, nonché i risultati positivi nelle manifestazioni comportamentali e nel benessere dei bambini/ragazzi.

5. **Ritiro dallo studio**

Lei ha il diritto di ritirare in qualsiasi momento il suo consenso alla partecipazione a questo studio, anche senza preavviso o motivazione specifica.

6. **Restituzione**

Lei ha diritto a richiedere informazioni sui risultati e sull'esito della ricerca

7. **Misure previste per tutelare l'anonimato**

I dati saranno raccolti in forma confidenziale (ad ogni nome viene associato un codice). In ogni caso i dati saranno utilizzati esclusivamente per scopi scientifici e statistici e nel rispetto della normativa vigente sulla protezione dei dati personali. I risultati della ricerca saranno pubblicati in forma riassuntiva e in nessun caso eventuali brevi citazioni saranno riconducibili a singole persone.

8. **Contatti**

Per qualsiasi informazione e chiarimento su questo studio o per qualsiasi necessità può rivolgersi ***al dott. Riccardo Lodi [per il gruppo di partecipanti residenti sul territorio di Parma] / alla dott.ssa Valentina Molin [per il gruppo di partecipanti residenti in Trentino]*** che sarà a sua disposizione per ulteriori informazioni o chiarimenti.

Consenso informato alla partecipazione alla ricerca

Il/la sottoscritto/a ____________ residente in __________, via _____

DICHIARA

- ☐ di aver letto il suddetto foglio informativo ricevuto, di aver compreso sia le informazioni in esso contenute sia eventuali informazioni fornite in forma orale dal personale addetto al progetto di ricerca *(indicare titolo del progetto*) e di aver avuto ampio tempo ed opportunità di porre domande ed ottenere risposte soddisfacenti dal personale addetto;
- ☐ di aver compreso che la partecipazione allo studio è del tutto volontaria e libera, che ci si potrà ritirare dallo studio in qualsiasi momento, senza dover dare spiegazioni e senza che ciò comporti alcuno svantaggio o pregiudizio;
- ☐ di aver compreso la natura e le attività che la partecipazione allo studio comportano e i relativi eventuali rischi;

- di aver compreso che la partecipazione a questo studio non comporterà il riconoscimento di alcun vantaggio di natura economica diretto o indiretto.

Conseguentemente, il/la sottoscritto/a
 ACCONSENTE NON ACCONSENTE

A partecipare allo studio, nella consapevolezza che tale consenso è manifestato liberamente ed è revocabile in ogni momento senza che ciò comporti alcuno svantaggio o pregiudizio.

Data ____________ *Firma*________________________________

CONSENSO INFORMATO ALLA PARTECIPAZIONE ALLA RICERCA- MINORI

1. **Titolo, Responsabile progetto**

Gentili Signori,
Vostra/o figlia/o , ovvero la/il minore sotto la Vostra tutela, è invitata/o a prendere parte a una ricerca condotta nel quadro del progetto "Validazione italiana con studio multicentrico RCT del progetto Family UNited per il rafforzamento delle competenze familiari", di cui è responsabile la dott.ssa Annalisa Pelosi, Dipartimento di Medicina e Chirurgia, Unità di Neuroscienze (Ateneo di Parma).

Prima di decidere in merito alla partecipazione è importante che abbiate tutte le informazioni necessarie per aderire in modo consapevole e responsabile. Vi chiediamo di leggere questo documento e di porre a chi Vi ha proposto questo studio tutte le domande che ritenete opportune.

2. **Breve descrizione e Obiettivi**

Il progetto di ricerca "Validazione italiana con studio multicentrico RCT del progetto Family UNited per il rafforzamento delle competenze familiari" persegue l'obiettivo di validare un progetto di miglioramento della coesione, della comunicazione e delle relazioni familiari, nonché delle abilità di regolazione emotiva e abilità assertive dei figli. Per raggiungere questo obiettivo, i ricercatori impegnati nel progetto si propongono di coinvolgere piccoli gruppi di genitori/caregiver e minori, di età compresa tra 8 e 15 anni, in quattro incontri a cadenza settimanale: nella prima ora di ogni incontro due facilitatori, opportunamente formativi, lavoreranno con i genitori/caregivers per imparare a conoscere il comportamento dei bambini e capire come l'attenzione del caregiver cambi il comportamento dei bambini, apprendere tecniche per facilitare la manifestazione dei comportamenti desiderati nei bambini e per comunicare con i loro figli promuovendone l'ascolto e la percezione di sentirsi sicuri e amati. In una diversa stanza, altri due facilitatori interagiranno con i bambini/ ragazzi, che apprenderanno modi positivi per affrontare lo stress e l'importanza di parlare dei propri sentimenti con adulti fidati, riflettendo sulle responsabilità di chi si prende cura di loro e delle qualità positive che vogliono sviluppare; inoltre, i bambini apprenderanno a gestire le situazioni di pressione da parte dei coetanei. Nella seconda ora, tutti i facilitatori, i genitori/ caregiver e i minori si riuniranno nella stessa stanza per la sessione familiare, in cui potranno mettere in pratica una comunicazione positiva e rispettosa, facendo giochi ed attività che incoraggiano una relazione positiva e creano fiducia.

3. **Che cosa comporta la partecipazione allo studio?**

La partecipazione al progetto di ricerca “Validazione italiana con studio multicentrico RCT del progetto Family UNited per il rafforzamento delle competenze familiari” comporta, oltre alla partecipazione alle quattro sedute del programma descritte nel punto precedente, la compilazione di test standardizzati: i minori compileranno scale di rilevazione della resilienza e dell’atteggiamento verso la .violenza interpersonale tra pari. Tutti gli strumenti saranno compilati online, prima di iniziare il progetto, dopo due e sei settimane dal termine del progetto.

4. **Benefici, disagi e/o rischi potenziali della partecipazione**

La partecipazione allo studio è volontaria e a titolo gratuito. Per i minori partecipanti la collaborazione non comporta nessun tipo di rischio o disagio. I ricercatori si attendono sul miglioramento delle competenze familiari e della qualità della vita relazionale ed emotiva della famiglia, nonché i risultati positivi nelle manifestazioni comportamentali e nel benessere dei bambini/ragazzi.

5. **Ritiro dallo studio**

Voi avete il diritto di ritirare in qualsiasi momento il consenso alla partecipazione di Vostra/o figlia/o, ovvero della/del minore sotto la Vostra tutela, da questo studio, anche senza preavviso o motivazione specifica.

6. **Restituzione**

Voi avete il diritto a richiedere informazioni sui risultati e sull’esito della ricerca.

7. **Misure previste per tutelare l’anonimato**

I dati saranno raccolti in forma confidenziale (ad ogni nome viene associato un codice). In ogni caso i dati saranno utilizzati esclusivamente per scopi scientifici e statistici e nel rispetto della normativa vigente sulla protezione dei dati personali. I risultati della ricerca saranno pubblicati in forma riassuntiva e in nessun caso eventuali brevi citazioni saranno riconducibili a singole persone.

8. **Contatti**

Per qualsiasi informazione e chiarimento su questo studio o per qualsiasi necessità può rivolgersi ***al dott. Riccardo Lodi, riccarlodi@gmail.com [per il gruppo di partecipanti residenti sul territorio di Parma] / alla dott.ssa Valentina Molin, valetinamolin@yahoo.it [per il gruppo di partecipanti residenti in Trentino]*** che sarà a sua disposizione per ulteriori informazioni o chiarimenti.

Consenso informato alla partecipazione alla ricerca

MADRE:

La sottoscritta ___________ residente in _________, via _____

DICHIARA

- ☐ di aver letto il suddetto foglio informativo ricevuto, di aver compreso sia le informazioni in esso contenute sia eventuali informazioni fornite in forma orale dal personale addetto al progetto di ricerca (*indicare titolo del progetto*) e di aver avuto ampio tempo ed opportunità di porre domande ed ottenere risposte soddisfacenti dal personale addetto;
- ☐ di aver compreso che la partecipazione allo studio è del tutto volontaria e libera, che ci si potrà ritirare dallo studio in qualsiasi momento, senza dover dare spiegazioni e senza che ciò comporti alcuno svantaggio o pregiudizio;
- ☐ di aver compreso la natura e le attività che la partecipazione allo studio comportano e i relativi eventuali rischi;

- di aver compreso che la partecipazione a questo studio non comporterà il riconoscimento di alcun vantaggio di natura economica diretto o indiretto.

Conseguentemente, la sottoscritta

 ACCONSENTE NON ACCONSENTE

A che la/il propria/o figlia/o partecipi allo studio, nella consapevolezza che tale consenso è manifestato liberamente ed è revocabile in ogni momento senza che ciò comporti alcuno svantaggio o pregiudizio.

Data ____________ *Firma*_______________________________

PADRE:

Il sottoscritto ___________ residente in _________, via _____

DICHIARA

- di aver letto il suddetto foglio informativo ricevuto, di aver compreso sia le informazioni in esso contenute sia eventuali informazioni fornite in forma orale dal personale addetto al progetto di ricerca *(indicare titolo del progetto*) e di aver avuto ampio tempo ed opportunità di porre domande ed ottenere risposte soddisfacenti dal personale addetto;
- di aver compreso che la partecipazione allo studio è del tutto volontaria e libera, che ci si potrà ritirare dallo studio in qualsiasi momento, senza dover dare spiegazioni e senza che ciò comporti alcuno svantaggio o pregiudizio;
- di aver compreso la natura e le attività che la partecipazione allo studio comportano e i relativi eventuali rischi;
- di aver compreso che la partecipazione a questo studio non comporterà il riconoscimento di alcun vantaggio di natura economica diretto o indiretto.

Conseguentemente, il sottoscritto

 ACCONSENTE NON ACCONSENTE

A che la/il propria/o figlia/o partecipi allo studio, nella consapevolezza che tale consenso è manifestato liberamente ed è revocabile in ogni momento senza che ciò comporti alcuno svantaggio o pregiudizio.

TUTORE / TUTRICE LEGALE:

Il sottoscritto ___________ residente in _________, via _____

DICHIARA

- di aver letto il suddetto foglio informativo ricevuto, di aver compreso sia le informazioni in esso contenute sia eventuali informazioni fornite in forma orale dal personale addetto al progetto di ricerca *(indicare titolo del progetto*) e di aver avuto ampio tempo ed opportunità di porre domande ed ottenere risposte soddisfacenti dal personale addetto;
- di aver compreso che la partecipazione allo studio è del tutto volontaria e libera, che ci si potrà ritirare dallo studio in qualsiasi momento, senza dover dare spiegazioni e senza che ciò comporti alcuno svantaggio o pregiudizio;

- ☐ di aver compreso la natura e le attività che la partecipazione allo studio comportano e i relativi eventuali rischi;
- ☐ di aver compreso che la partecipazione a questo studio non comporterà il riconoscimento di alcun vantaggio di natura economica diretto o indiretto.

Conseguentemente, il sottoscritto

☐ ACCONSENTE ☐ NON ACCONSENTE

A che la / il minore sotto la propria tutela partecipi allo studio, nella consapevolezza che tale consenso è manifestato liberamente ed è revocabile in ogni momento senza che ciò comporti alcuno svantaggio o pregiudizio.

Data ____________ *Firma*________________________________

Participant information sheet for the interviews in English

Assessing the effectiveness and acceptability of Family UNited – Italy

Participant Information Sheet-Caregiver

You are being invited to take part in a research study which aims to examine your experience after taking part in the Family UNited Programme. Before you decide it is important for you to understand why the research is being done and what it will involve. Please take time to read the following information carefully and discuss it with others if you wish. Please ask if there is anything that is not clear or if you would like more information. Take time to decide whether or not you wish to take part. Thank you for reading this.

Who will conduct the research?

The research will be conducted by a team of researchers from The United Nations Office on Drugs and Crime (UNODC) in collaboration with the University of Parma and the University of Trento.

Title of the Research

Assessing the effectiveness and acceptability of Family UNited in Trento and Parma, Italy..

What is the aim of the research?

This study wants to examine the views and experiences of those parents that took part in the Family UNited Programme

Why have I been chosen?

You have been chosen because you took part in the Family UNited Programme.

What would I be asked to do if I took part?

You will take part in an individual interview. The interview lasts about 1 hour and the main areas to be covered are: 1) overall parenting experience, 2) effectiveness of the program and 3) cultural relevance of the program.

What happens to the data collected?

The data collected will be stored confidentially for a period of 5 years. The interviews will be transcribed and then analysed.

How is confidentiality maintained?

The information that you provide will not be shared with anyone outside the research team. What you say during the interviews will be audio-recorded. Audio recordings will be deleted as soon as they are transcribed. Your name and any other personal information will not be included in transcriptions. All paper and digital data will be stored securely. At the end of the study, the information will be anonymised.

It is important for you to know that in case you disclose child abuse or domestic violence, relevant authorities will need to be informed.

What happens if I do not want to take part or if I change my mind?

It is up to you to decide whether or not to take part. If you do decide to take part you will be given this information sheet to keep and be asked to sign a consent form. If you decide to take part you are still free to withdraw at any time without giving a reason and without detriment to yourself. You will have 7 days after the interview to withdraw your interview from the research.

Will I be paid for participating in the research?

No. You will not be paid for taking part on this research.

What is the duration of the research?

The interview will last 1 hour.

Where will the research be conducted?

The research will be conducted in the same place that the Family UNited Programme was running.

Will the outcomes of the research be published?

Yes. They will be published in journal articles and in conference presentations.

Contact for further information

Parma: Riccardo Lodi, riccarlodi@gmail.com

Trentino: Valentina Molin, valentina.molin@unitn.it

What if I have further questions about my child?

If you need further advice regarding your child behaviour, you should contact your child's teacher or the school counsellor. You might also wish to look for advice at the community health centre.

Interview guide in English

Family UNited - Italy

Schedule for process evaluation interview with parents/carers

Before interview:

Check that the participant understands the purpose of the interview
Ensure informed consent has been obtained, check permission to record the interview
Provide assurances regarding confidentiality
Remind the participant that they can stop the interview at any time and leave, or ask questions
Provide them with an opportunity to ask questions before starting

1. Before Family UNited

- How did you hear about the study?
- How did you feel about taking part in the programme? (reluctant/eager/worried-explore if any barriers mentioned)
- Have you taken part or heard of other similar programmes?

2. During Family UNited

- What made you keep coming to the sessions?
- What did you think about the programme?:
    - the activities (parent / family sessions)
    - facilitators
    - being in the group
    - the knowledge you gained
- Do you think that programme was culturally acceptable for you/ were the activities suitable for your beliefs and traditions?
- What did your children think of the activities?
- If you could change anything about Family UNited, what would it be?

3. After Family UNited

- What happened then?
    - Changes in family
    - Contact with others who took part
- Is there anything else like Family UNited currently available to you?

4. And finally..

- What's the best thing about Family UNited?

Participant questionnaires in English

FDQ

**Family Identification Number**	

Family Demographics Questionnaire

The questionnaire that we ask you to fill out is included in the Family UNited Project in which you are participating with your son / daughter: your answers will be used to evaluate the its effectiveness. In this way, you will also help the Project to develop new and increasingly effective initiatives for parents like you. There are no right or wrong answers: choose the one that best reflects your thinking. All your answers will be anonymous: the code we ask you to enter has the sole purpose of making the processing of the answers more accurate, allowing us to link your answers before and after our meetings

1. Year of birth: ………

2. The gender you identify with: •Male • Female • Not binary

3. Your current marital status: • Married • Divorced/separated • Single • Widow/er

4. You are living with:
- Partner and children • Only partner • Only children • Partner, children and other
- Children and other relatives • Parner and other relatives • Other: ___________________________

5. Your highest level of education:
- primary school or less • some high school • completed high school
- trade/technical college qualification • university degree or post-graduate degree

5. Your partner's highest level of education (if applicable)?
- primary school or less • some high school • completed high school
- trade/technical college qualification • university degree or post-graduate degree

6. Are you working outside the home right now?
   - yes, full time • yes, part time • not working, but looking for a job
   - home based paid work (child care, sewing, internet or phone-based work, etc)
   - not working (includes stay at home parents, retired)

7. Is your partner working outside the home right now (if applicable)?
   - yes, full time • yes, part time • not working, but looking for a job
   - home based paid work (child care, sewing, internet or phone-based work, etc)
   - not working (includes stay at home parents, retired)

8. How many children do you care for aged 0-15? ....

9. Insert details for the main child taking part in this programme with you

Age Today	Gender	Your relationship to Child (e.g parent, step-parent, aunt..)

10. Please provide further details about each of your OTHER children in the table.

Child	Age Today	Gender	Your relationship to Child (e.g parent)
1.			
2.			
3.			
4.			
5.			
6.			

11a. Is your country of origin Italy ? Please tick

**Yes**	
**No**<br><br>11b. If your country of origin is not Italy, how long have you and your child (taking part in the study) been living in Italy?<br><br>……Years ……Months	

SDQ

Family Identification Number			
Please Circle:	Time 1<br><br>(pre-measure)	Time 2<br><br>(2 weeks post program)	Time 3<br><br>(6 weeks post program)

Strengths and Difficulties Questionnaire

For each item, please mark the box for Not True, Somewhat True or Certainly True on the table. It would help us if you answered all items as best you can even if you are not absolutely certain or the item seems daft! Please give your answers on the basis of the child's behaviour that took part in the Family UNited Programme with you.

	**Not true**	**Some-what true**	**Certainly true**
Considerate of other people's feelings (1)			
Restless, overactive, cannot stay still for long (2)			
Often complains of headaches, stomach-aches or sickness (3)			
Shares readily with other children (treats, toys, pencils etc.) (4)			
Often has temper tantrums or hot tempers (5)			
Rather solitary, tends to play alone (6)			
Generally obedient, usually does what adults request (7)			
Many worries, often seems worried (8)			
Helpful if someone is hurt, upset or feeling ill (9)			
Constantly fidgeting or squirming (10)			
Has at least one good friend (11)			
Often fights with other children or bullies them (12)			
Often unhappy, down-hearted or tearful (13)			
Generally liked by other children (14)			
Easily distracted, concentration wanders (15)			
Nervous or clingy in new situations, easily loses confidence (16)			
Kind to younger children (17)			

Often lies or cheats (18)			
Picked on or bullied by other children (19)			
Often volunteers to help others (parents, teachers, other children) (20)			
Thinks things out before acting (21)			
Steals from home, school or elsewhere (22)			
Gets on better with adults than with other children (23)			
Many fears, easily scared (24)			
Sees tasks through to the end, good attention span (25)			

PAFAS

Family Identification Number			
Please Circle:	Time 1<br><br>(pre-measure)	Time 2<br><br>(2 weeks post program)	Time 3<br><br>(6 weeks post program)

Parenting and Family Adjustment Scales (PAFAS)

(Sanders, Morawska, Filus & Fletcher)

The rating scale is as follows:

0. Not true of me at all

1. True of me a little, or some of the time

2. True of me quite a lot, or a good part of the time

3. True of me very much, or most of the time

How true is this of you?		Not at all	A little	Quite a lot	Very much
1	If my child doesn't do what they're told to do, I give in and do it myself	0	1	2	3
2	I give my child a treat, reward or fun activity for behaving well	0	1	2	3
3	I follow through with a consequence (e.g. take away a toy) when my child misbehaves	0	1	2	3
4	I threaten something (e.g. to turn off TV) when my child misbehaves but I don't follow through	0	1	2	3
5	I shout or get angry with my child when they misbehave	0	1	2	3
6	I praise my child when they behave well	0	1	2	3
7	I try to make my child feel bad (e.g. guilt or shame) for misbehaving to teach them a lesson	0	1	2	3
8	I give my child attention (e.g. a hug, wink, smile or kiss) when they behave well	0	1	2	3
9	I spank (smack) my child when they misbehave	0	1	2	3
10	I argue with my child about their behaviour/attitude	0	1	2	3
11	I deal with my child's misbehaviour the same way all the time	0	1	2	3
12	I give my child what they want when they get angry or upset	0	1	2	3
13	I get annoyed with my child	0	1	2	3

14	I chat/talk with my child	0	1	2	3
15	I enjoy giving my child hugs, kisses and cuddles	0	1	2	3
16	I am proud of my child	0	1	2	3
17	I enjoy spending time with my child	0	1	2	3
18	I have a good relationship with my child	0	1	2	3
19	I feel stressed or worried	0	1	2	3
20	I feel happy	0	1	2	3
21	I feel sad or depressed	0	1	2	3
22	I feel satisfied with my life	0	1	2	3
23	I cope with the emotional demands of being a parent	0	1	2	3
24	Our family members help or support each other	0	1	2	3
25	Our family members get on well with each other	0	1	2	3
26	Our family members fight or argue	0	1	2	3
27	Our family members criticize or put each other down	0	1	2	3

**If you are in the relationship please answer the following 3 questions**		**Not at all**	**A little**	**Quite a lot**	**Very much**
28	I work as a team with my partner in parenting	0	1	2	
29	I disagree with my partner about parenting	0	1	2	3
30	I have a good relationship with my partner	0	1	2	3

CYRM-R

**Family Identification Number**			
**Please Circle:**	**Time 1**<br><br>**(pre-measure)**	**Time 2**<br><br>**(2 weeks post program)**	**Time 3**<br><br>**(6 weeks post program)**

PMK-CYRM-R

To what extent do the following statements apply to the child you are caring for that is taking part in the intervention with you? Remember to keep the same child in mind for all questions. There are no right or wrong answers

		Not at all.<br>[1]<br>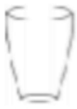	A little<br><br>[2]<br>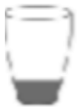	Some what<br>[3]<br>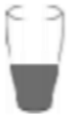	Quite a bit<br>[4]<br>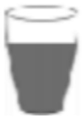	A lot<br><br>[5]<br>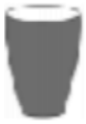
1	They cooperate with people around them					
2	They believe getting an education or doing well in school is important to them					
3	They know how to behave/act in different situations (like school, home)					
4	They have a parent(s)/caregiver(s) who knows where they are and what they are doing most of the time					
5	They have a parent(s)/caregiver(s) that know a lot about them (for example what makes them happy or sad, scared)					
6	They have enough to eat at their home when they are hungry					
7	They are fun to be with or that others like to play with them					
8	They talk to their family/caregiver(s) about how they feel					
9	They feel supported by their friends					
10	They feel that they fit in at their school					
11	They have a family/caregiver(s) who cares about them when times are hard (if they are sick or have done something wrong)					
12	They have friends who care about them when times are hard (if they are sick or have done something wrong)					

13	They are treated fairly					
14	They are given chances to show others that they are growing up and can do things by themselves					
15	They feel safe when they are with their family/caregiver(s)					
16	They have chances to learn things that will be useful when they are older (like cooking, working and helping others)					
17	They like the ways their family/caregiver(s) celebrates things (like holidays or learning about their cultural)					

ATIPV

Family Identification Number			
Please Circle:	Time 1<br><br>(pre-measure)	Time 2<br><br>(2 weeks post program)	Time 3<br><br>(6 weeks post program)

1. If I walked away from a fight, I'd be a coward ("chicken").
   ☐ Disagree a lot ☐ Disagree a little ☐ Agree a little ☐ Agree a lot

2. The best way to stop a fight before it starts is to stop the argument (problem) that caused it.
   ☐ Disagree a lot ☐ Disagree a little ☐ Agree a little ☐ Agree a lot

3. Anyone who won't fight is going to be "picked on" even more.
   ☐ Disagree a lot ☐ Disagree a little ☐ Agree a little ☐ Agree a lot

4. I don't need to fight because there are other ways to deal with being mad.
   ☐ Disagree a lot ☐ Disagree a little ☐ Agree a little ☐ Agree a lot

5. It's OK to hit someone who hits you first.
   ☐ Disagree a lot ☐ Disagree a little ☐ Agree a little ☐ Agree a lot

6. If my friends want to go someplace where a fight might happen, I find it easy to say I don't want to go with them.
   ☐ Disagree a lot ☐ Disagree a little ☐ Agree a little ☐ Agree a lot

7. When actions of others make me angry, I can usually deal with it without getting into a physical fight.
   ☐ Disagree a lot ☐ Disagree a little ☐ Agree a little ☐ Agree a lot

8. If a kid teases me or "disses" me, I usually cannot get them to stop unless I hit them.
   ☐ Disagree a lot ☐ Disagree a little ☐ Agree a little ☐ Agree a lot

9. If a kid at school hits me, it is harder to report them to a teacher or other adult than it is to just hit them back.
   ☐ Disagree a lot ☐ Disagree a little ☐ Agree a little ☐ Agree a lot

10. If I really want to, I can usually talk someone out of trying to fight with me.
    ☐ Disagree a lot ☐ Disagree a little ☐ Agree a little ☐ Agree a lot

11. My family would be mad at me if I got in a fight with another student, no matter what the reason.
    ☐ Disagree a lot ☐ Disagree a little ☐ Agree a little ☐ Agree a lot

12. If a student hits me first, my family would want me to hit them back.
    ☐ Disagree a lot ☐ Disagree a little ☐ Agree a little ☐ Agree a lot

13. I usually can tell when things are bothering me or getting on my nerves.
    ☐ Disagree a lot ☐ Disagree a little ☐ Agree a little ☐ Agree a lot

14. If things are bothering me or getting on my nerves, I do things to relax.
    ☐ Disagree a lot ☐ Disagree a little ☐ Agree a little ☐ Agree a lot

Participant questionnaires in Italian

Questionario demografico familiare

Gentilissima, Gentilissimo,

il questionario che ti chiediamo di compilare è previsto all'interno del Progetto Family UNited cui stai partecipando con tuo figlio / tua figlia: le tue risposte serviranno a valutare l'efficacia del percorso svolto insieme. In questo modo, aiuterai anche il Progetto a sviluppare iniziative sempre nuove e sempre più efficaci per genitori come te. Non ci sono risposte giuste o sbagliate: scegli quella che rispecchia meglio il tuo pensiero. Tutte le tue risposte saranno anonime: il codice che ti chiediamo di inserire ha il solo scopo di rendere più accurata l'elaborazione delle risposte, permettendoci di collegare le tue risposte prima e dopo i nostri incontri in classe.

Alcune domande su di te e sulla tua famiglia
Innanzitutto, ti chiediamo di rispondere ad alcune domande su di te e sulla tua famiglia.

1. Il tuo anno di nascita: ______
2. Il genere in cui ti identifichi: ☐ Maschio ☐ Femmina ☐ Non binario
3. Stato civile : ☐ Sposato/a ☐ Divorziato/a o separato/a ☐ Libero/a ☐ Vedovo/a
4. Condizione abitativa. Vivi con:
   ☐ Partner e figli ☐ Solo partner ☐ Solo figli ☐ Partner, figli e altri familiari
   ☐ Figli e altri familiari ☐ Partner e altri familiari ☐ Altro: _______________________
5. Titolo di studio:
   ☐ Nessun titolo/ licenza elementare ☐ Licenza scuola media inferiore
   ☐ Qualifica professionale ☐ Diploma scuola superiore ☐ Laurea o titolo superiore
6. Titolo di studio del tuo partner (se applicabile):
   ☐ Nessun titolo/ licenza elementare ☐ Licenza scuola media inferiore
   ☐ Qualifica professionale ☐ Diploma scuola superiore ☐ Laurea o titolo superiore
7. Attualmente lavori fuori casa?
   ☐ sì, a tempo pieno ☐ sì, part-time ☐ non sto lavorando ma sto cercando un lavoro
   ☐ lavoro da casa retribuito (assistenza all’infanzia, sartoria, lavoro con internet o con telefono, ecc.)
   ☐ non lavoro (incluso vivere con i genitori, essere in pensione)
8. Il tuo partner attualmente lavora fuori casa? (se applicabile)
   ☐ sì, a tempo pieno ☐ sì, part-time ☐ non sto lavorando ma sto cercando un lavoro
   ☐ lavora da casa retribuito (assistenza all’infanzia, sartoria, lavora con internet o con telefono, ecc.)
   ☐ non lavora (incluso vivere con i genitori, essere in pensione)
9. Di quanti bambini ti prendi cura? ____

Per favore, fornisci ulteriori dettagli su ciascuno dei bambini nella tabella seguente.

Bambino	Età	Genere	Qual è la relazione tra voi (es: tuo/a figlio/a, figlio/a del partner, nipote, etc.)

1.			
2.			
3.			
4.			
5.			

10. Qual è il tuo paese d'origine? ____
11. Se alla domanda precedente hai indicato un Paese diverso dall'Italia, da quanto tempo vivi qui? ____
12. Se tuo figlio (quello che partecipa al progetto con te) non è nato in Italia, da quanto tempo vive qui? ___
13. Con chi vivete ora tu e tuo figlio (partecipante allo studio)?
____

Punti di forza e criticità di tuo figlio/ tua figlia

[Strenght and Difficulties Questionnaire - SDQ; Marzocchi, G.M., Di Pietro M., Vio, C., Bassi, E.Filoramo, G., Dalmaso, A., 2002]

Ora vorrei farti alcune domande relativamente a tuo figlio / tua figlia (quello/a che partecipa con te al progetto Family UNited). Per ogni affermazione segna se la ritieni "Non vera", "Abbastanza vera" o "Assolutamente vera". Ci aiuterebbe se rispondessi a tutte le voci nel miglior modo possibile, anche se non sei assolutamente certo/a o l'affermazione ti sembra stupida! Dai le tue risposte sulla base del comportamento del bambino/ragazzo che partecipa al Programma Family UNited con te.

	**Non vero**	**Abbastanza vero**	**Vero**
Rispettoso/a dei sentimenti altrui	•	•	•
Irrequieto/a, iperattivo/a, incapace di stare fermo/a per molto tempo	•	•	•
Si lamenta spesso per mal di testa, mal di stomaco o nausea	•	•	•
Condivide volentieri con gli altri bambini (dolci, giocattoli, matite ecc.)	•	•	•
Spesso ha crisi di collera o è di cattivo umore	•	•	•
Piuttosto solitario/a, tende a giocare/stare da solo	•	•	•
È generalmente obbediente, esegue di solito le richieste degli adulti	•	•	•
Ha molte preoccupazioni, spesso sembra preoccupato/a	•	•	•
È d'aiuto se qualcuno si fa male, è arrabbiato o malato	•	•	•
Costantemente in movimento o a disagio	•	•	•
Ha almeno un buon amico o una buona amica	•	•	•

Spesso litiga con gli altri bambini/ragazzi o li infastidisce di proposito	•	•	•
Spesso infelice, triste o in lacrime	•	•	•
Generalmente ben accettato dagli altri bambini/ragazzi	•	•	•
Facilmente distratto, incapace di concentrarsi	•	•	•
È nervoso/a o a disagio in situazioni nuove, si sente poco sicuro/a di sé	•	•	•
Gentile con i bambini/ragazzi più piccoli	•	•	•
Spesso dice bugie o inganna	•	•	•
Preso/a di mira o preso in giro dagli altri bambini/ragazzi	•	•	•
Si offre spesso volontario/a per aiutare gli altri (genitori, insegnanti, altri bambini/ragazzi)	•	•	•
Pensa prima di fare qualcosa	•	•	•
Ruba a casa, a scuola o in altri posti	•	•	•
Ha rapporti migliori con gli adulti che con i bambini/ragazzi	•	•	•
Ha molte paure, si spaventa facilmente	•	•	•
È in grado di portare a termine ciò che gli viene richiesto, rimanendo concentrato per tutto il tempo necessario	•	•	•

Scale di adattamento dei genitori e della famiglia

[Parenting and Family Adjustment Scales – PAFAS; Sanders, Morawska, Filus & Fletcher, 2014]

Ora vorrei chiederti alcune cose rispetto al rapporto con tuo figlio (quello che partecipa con te al Progetto Family UNited) e al clima che c'è in famiglia. Per ogni affermazione proposta ti chiedo di rispondere seguendo la seguente scala di valutazione:

- Per niente vero per me
- Un po' vero per me, o qualche volta vero
- Abbastanza vero per me, o per una buona parte del tempo vero
- Molto vero per me, o per la maggior parte del tempo vero

	**Per niente vero**	**Un po' vero, o qualche volta vero**	**Abbastanza vero, o per buona parte del tempo vero**	**Molto vero, o per la maggior del tempo vero**
Se mio figlio/a non fa quello che gli si dice, mi arrendo e lo faccio io	•	•	•	•
Ricompenso mio figlio/a con un regalo, un premio o un'attività divertente se si comporta bene	•	•	•	•
Punisco mio figlio/a (per esempio togliendogli un giocattolo) (mettere anche cellulare) quando si comporta male	•	•	•	•
Minaccio di fare qualcosa (per esempio di spegnere la TV) (mettere un altro esempio, tipo non uscire con gli amici) quando mio figlio si comporta male, ma poi non lo faccio	•	•	•	•

Grido o mi arrabbio con mio figlio/a quando si comporta male	•	•	•	•
Elogio mio figlio/a quando si comporta bene	•	•	•	•
Cerco di far sentire male mio figlio/a (per esempio facendogli provare senso di colpa o vergogna) per essersi comportato/a male per dargli una lezione	•	•	•	•
Do attenzioni a mio figlio/a (per esempio un abbraccio, faccio un occhiolino, un sorriso o gli do un bacio) quando si comporta bene	•	•	•	•
Sculaccio (do un ceffone) a mio figlio/a quando si comporta male	•	•	•	•
Discuto con mio figlio/a sul suo comportamento/atteggiamento	•	•	•	•
Affronto il comportamento sbagliato di mio figlio/a sempre allo stesso modo	•	•	•	•
Do a mio figlio/a ciò che vuole quando si arrabbia o si agita	•	•	•	•
Mi arrabbio con mio figlio/a	•	•	•	•
Chiacchiero/parlo con mio figlio/a	•	•	•	•
Mi piace abbracciare mio figlio/a, dargli baci e fargli le coccole	•	•	•	•
Sono orgoglioso/a di mio figlio/a	•	•	•	•
Mi piace passare il tempo con mio figlio/a	•	•	•	•
Ho un buon rapporto con mio figlio/a	•	•	•	•
Mi sento stressato/a o preoccupato/a	•	•	•	•
Mi sento felice	•	•	•	•
Mi sento triste o depresso/a	•	•	•	•
Mi sento soddisfatto/a della mia vita	•	•	•	•
Riesco a gestire le implicazioni emotive dell’essere un genitore	•	•	•	•
In famiglia ci aiutiamo o sosteniamo a vicenda	•	•	•	•
In famiglia andiamo d’accordo tra di noi	•	•	•	•
In famiglia discutiamo o litighiamo	•	•	•	•
In famiglia ci critichiamo o umiliamo a vicenda	•	•	•	•

Se sei in una relazione, rispondi alle seguenti domande:

	**Per niente vero**	**Un po’ vero, o qualche volta vero**	**Abbastanza vero, o per buona parte del tempo vero**	**Molto vero, o per la maggior del tempo vero**
Collaboro con il mio partner nella genitorialità	•	•	•	•

Non sono d'accordo con il mio partner nel modo di fare il genitore	•	•	•	•
Ho un buon rapporto con il mio partner	•	•	•	•

STRUMENTI PER I MINORI

Ciao,
ti chiediamo di rispondere a qualche domanda su di te e sulle persone che ti sono più vicine. Te lo chiediamo perché stai partecipando al Progetto Family UNited e le tue risposte saranno importantissime per sapere se siamo stati utili a te e alle persone cui vuoi bene. In questo modo, ci aiuterai a essere sempre più bravi ad aiutare ragazzi e ragazze come te a stare sempre meglio.
Per cominciare, è necessario che un tuo genitore / tutor ti autorizzi. Per favore, chiedigli / chiedile di leggere il paragrafo che segue e di indicare se è d'accordo che tu risponda al questionario

[Il consenso informato e l’informativa sulla privacysono qui omessi]

Il Progetto Family UNited che stai per iniziare si svolge: ¨ a Parma ¨ in Trentino

1. Quanti anni hai? ______
2. Sei: ¨ Maschio ¨ Femmina ¨ Preferisco non rispondere

Child and Youth Resilience Measure - CYRM
[Ungar et al., 2008]

Indica quanto ciascuna di queste affermazioni descrive la persona che sei, il modo in cui ti comporti o in cui pensi. Non ci sono risposte giuste o sbagliate: per noi è importante sapere come sei e cosa pensi.

	**Per niente**	**Poco**	**Un po’**	**Abbastanza**	**Molto**
Collaboro con le persone che mi circondano	•	•	•	•	•
Ricevere un’educazione è importante per me	•	•	•	•	•
So come comportarmi adeguatamente nelle diverse in situazioni	•	•	•	•	•
I miei genitori/tutori si prendono davvero cura di me	•	•	•	•	•
I miei genitori/tutori mi conoscono molto bene	•	•	•	•	•
Alle persone piace passare del tempo con me	•	•	•	•	•
Parlo con la mia famiglia/con i miei tutori di come mi sento	•	•	•	•	•
Mi sento supportato dai miei amici	•	•	•	•	•
Sento che sono parte della mia scuola	•	•	•	•	•
La mia famiglia/i miei tutori mi stanno vicino nei momenti difficili	•	•	•	•	•
I miei amici mi stanno vicino nei momenti difficili	•	•	•	•	•

Mi sento ben inserito nel mio quartiere / nel mio paese	•	•	•	•	•
Ho l'opportunità di dimostrare agli altri che sto diventando un adulto e posso comportarmi responsabilmente	•	•	•	•	•
Mi sento al sicuro quando sono con la mia famiglia/con i miei tutori	•	•	•	•	•
Ho l'opportunità di sviluppare competenze che saranno utili nel futuro (competenze sul lavoro e capacità di prendermi cura degli altri)	•	•	•	•	•
Mi piacciono le tradizioni culturali e familiari della mia famiglia/dei miei tutori	•	•	•	•	•

Attitude toward interpersonal peer violence - ATIPV

[Slaby, 1989]

	**Per niente d'accordo**	**Poco d'accordo**	**Abbastanza d'accordo**	**Molto d'accordo**
Se mi allontanassi da una rissa, sarei un vigliacco (un "pollo")	•	•	•	•
Il modo migliore di fermare una rissa prima che inizi è quello di fermare il motivo (problema) che l'ha causata	•	•	•	•
Chiunque non voglia battersi sarà preso di mira anche di più	•	•	•	•
Non ho bisogno di battermi, perché ci sono altri modi per esprimere la rabbia	•	•	•	•
È giusto colpire qualcuno che ti ha colpito per primo	•	•	•	•
Se i miei amici vogliono andare in qualche posto in cui potrebbe capitare una rissa, mi è facile dire che non voglio andare con loro	•	•	•	•
Quando le azioni degli altri mi fanno arrabbiare, di solito riesco a gestire la situazione senza battermi fisicamente	•	•	•	•
Se un bambino / ragazzo mi provoca o mi offende, di solito non riesco a farlo smettere se non lo picchio	•	•	•	•
Se un bambino/un ragazzo a scuola mi colpisce, è più difficile dirlo a un insegnante o un altro adulto che restituirgli il colpo	•	•	•	•
Se davvero lo voglio, di solito posso far cambiare idea a qualcuno che vuole battersi con me	•	•	•	•
La mia famiglia si arrabbierebbe con me se mi trovassi coinvolto in una rissa con altro studente, non importa per quale motivo	•	•	•	•
Se uno studente mi colpisse per primo, la mia famiglia vorrebbe che gli restituissi il colpo	•	•	•	•
Di solito riesco a dire quando le cose mi infastidiscono o mi irritano	•	•	•	•

Se succedono cose che mi infastidiscono o mi irritano, faccio qualcosa per rilassarmi	•	•	•	•
